# Parathyroid Gland Involvement by Thyroid Cancer: Results from a Large Series of Thyroidectomies Performed in Two Italian University Hospitals and Review of the Literature

**DOI:** 10.1155/2014/685425

**Published:** 2014-11-11

**Authors:** Giampaolo Papi, Stefania Corrado, Guido Fadda, Antonino Maiorana, Livia Maccio, Salvatore Maria Corsello, Alfredo Pontecorvi

**Affiliations:** ^1^Institute of Endocrinology, Catholic University of Rome, 00168 Rome, Italy; ^2^Institute of Anatomic Pathology, University of Modena and Reggio Emilia, 41100 Modena, Italy; ^3^Institute of Anatomic Pathology, Catholic University of Rome, 00168 Rome, Italy

## Abstract

*Objectives.*Parathyroid involvement by thyroid cancer (TC) has not been frequently investigated in thyroidectomy-based studies. We aimed to detect cases of parathyroid invasion by TC in a large series of thyroidectomies and to review the literature on this topic.* Study Design. *A 10-yr period database research was made from the files of the Section of Pathology of two Italian University Hospitals. Out of 22,310 thyroidectomies, 10 patients with parathyroid involvement by TC were found.* Results. *The 10 patients, 7 females and 3 males, aged 55 ± 14 years (range 34–76, median 56) had papillary thyroid carcinoma and accounted for 0.4% of subjects affected by all TCs and submitted to thyroidectomy. The tumor invaded perithyroid soft tissues in 6 patients and central neck (level VI) lymph nodes in 3. Parathyroid involvement by TC occurred by infiltration in 6 cases, extension through an intervening pseudocapsule in 1, and both patterns in 3. All patients are alive and disease free at 5.6 ± 3-yr follow-up.* Conclusion.* Limited to thyroidectomy series, our results and literature data suggest that parathyroid involvement by TC has a 0.4–3.9% incidence rate; mainly affects women in their sixth-seventh decade of life; is associated to a good prognosis, unless massive extrathyroid extension of TC occurs.

## 1. Introduction

Thyroid cancer (TC) represents a rare event, accounting for almost 1% of all human tumors [1]. Papillary thyroid carcinoma (PTC) is by far the most common TC, as it represents approximately 85% of primary thyroid neoplasms [2]. Usually, PTC shows a nonaggressive behavior and carries an excellent prognosis, with a 10-yr survival rate exceeding 90% [1, 2]. Nonetheless, spread to locoregional lymph nodes occurs in up to 70% of PTC cases [3], whereas distant metastases develop rarely.

Owing to their anatomic location, adjacent to the thyroid, parathyroid glands should be at high risk of tumor involvement in patients with TC. Unexpectedly, only four thyroidectomy-based series of adult patients have been as yet reported in the literature [4–7], describing parathyroid invasion by TC, of which three originated from the same institution, the Kuma Hospital in Japan [4–6].

To the best of our knowledge, no review has thus far been published in the literature focusing on the parathyroid involvement by primary TC.

Here, we report a series of patients submitted to thyroidectomy and diagnosed with parathyroid invasion by PTC and discuss related clinical, pathological, and follow-up features. Furthermore, we review the published literature on this topic.

## 2. Patients and Methods

### 2.1. Study Design

To detect cases of parathyroid invasion by TC, a database research having “histology, papillary thyroid carcinoma, follicular thyroid carcinoma, Hurthle cell thyroid carcinoma, medullary thyroid carcinoma, anaplastic thyroid carcinoma, parathyroid, and metastasis” as keywords and spanning the period from January 2003 to December 2013, was made from the files of the Sections of Pathology of both the Catholic University of Rome and the University of Modena and Reggio Emilia, Italy.

Once cases of TC metastases to parathyroid were selected, the following data were retrospectively sought: age; sex; reports of cytology and histological examinations; reports of radioiodine remnant ablation (I^131^); results of recombinant human thyrotropin stimulation test; follow-up time.

PubMed was searched for parathyroid gland involvement by TC, using a combination of the following search terms: “parathyroid,” “thyroid,” “neoplasm,” “cancer,” “papillary thyroid carcinoma,” “follicular thyroid carcinoma,” “medullary thyroid carcinoma,” “anaplastic thyroid carcinoma,” and “metastasis.”

### 2.2. Patients

During the 10-year period of the study, 22,310 thyroidectomies were performed in our institutions. A total of 13 patients with parathyroid metastasis from TC (11 papillary thyroid carcinoma and 2 anaplastic thyroid carcinoma) were found. However, only 10 subjects were definitively included in the study, because data obtained from 3 patients were incomplete and unsatisfactory. All these 10 patients had been submitted to total thyroidectomy and lymph adenectomy of the central neck (level VI) compartment. Postsurgical complications were recorded in 2 patients, who both developed transient recurrent laryngeal nerve palsy.

### 2.3. Methods

Fine needle aspiration biopsy (FNAB) slides and the microscopic sections of thyroidectomy of the 10 patients enrolled in the study were reviewed.

FNAB was ultrasound-guided: multiple direct smears were prepared and the alcohol-fixed samples were stained by the Hematoxylin and Eosin method in the Section of Pathology of University Hospital of Modena and Reggio Emilia and by Papanicolaou in the Section of Pathology of the Catholic University of Rome.

The system used for reporting FNAB specimens was the 5-tier category classification (Thy 1 to Thy 5) proposed by the British Association—Royal College of Physicians in 2002 [8, 9] and modified by the Italian Society of Pathology and Cytopathology—Italian Section of the International Academy of Pathology in 2007 [10]. The 5 categories are the following: Thy 1 = non diagnostic; Thy 2 = benign lesion; Thy 3 = follicular neoplasm; Thy 4 = suspicious for malignancy; Thy 5 = malignant lesion.

All surgical specimens were fixed overnight in 10% neutral buffered formalin and paraplast-embedded. For histological examination, 5 micron thick sections were cut and stained with Hematoxylin and Eosin. Pathological stage report was based on AJCC/International Union against Cancer (AJCC/UICC) classification system [11]. According to previous literature [4–7], parathyroid involvement by TC was defined as follows: (1) direct invasion by infiltrative growth from the primary TC (pattern A); (2) extension of TC into the gland, from which cancer nests are separated by an intervening fibrous capsule (pseudocapsule) (pattern B); (3) true metastasis within the gland, with no evidence of continuity from the primary TC (pattern C).

After surgery, all 10 patients underwent radioactive iodine (RAI) remnant ablation following withdrawal of L-thyroxine substitution therapy. The risk level for TC recurrence was assigned in accordance with the recent American Thyroid Association guidelines [12].

Post-RAI surveillance for residual/recurrent TC was performed on l-thyroxine suppressive therapy, by using recombinant human thyrotropin (rhTSH) stimulation test (Thyrogen) 0.9 mg, administered i.m. for 2 consecutive days. Blood samples for measurement of serum TSH, thyroglobulin, and anti-thyroglobulin antibody concentrations were collected before the first rhTSH injection and 3 days and 5 days after the last injection of rhTSH. Serum TSH was measured using a commercial immunometric assay (Architect, Abbott Lab., Abbott Park IL, USA) with normal values ranging from 0.35 to 4.94 mcIU/mL. Serum thyroglobulin and anti-thyroglobulin antibodies were measured using a commercial immunometric assay (DXI800, Beckman Coulter inc., Fullerton CA, USA) with a lower detection limit of 0.1 ng/mL and 4 IU/mL, respectively. Patients with undetectable stimulated thyroglobulin on rhTSH stimulation test were followed yearly by either measurement of serum thyroglobulin and anti-thyroglobulin antibody concentrations and neck ultrasound.

Results are reported as mean ± standard deviation (SD).

## 3. Results

Of 22,310 cases of thyroidectomy, 2,753 (12.3%) primary TC were found, of which 2,560 (93%) were PTC, 110 (4%) follicular thyroid carcinomas, 55 (2%) medullary thyroid carcinomas, 15 (0.6%) anaplastic thyroid carcinomas (ATC), and 13 (0.4%) metastases from other organs. In 10 of these malignancies, a concurrent parathyroid involvement was detected, accounting for 0.4% of subjects affected by all primary TC.

**Figure 1:**
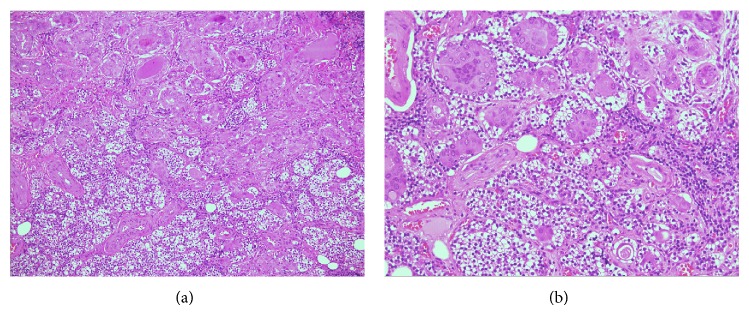
Papillary thyroid carcinoma, tall cell variant. The tumour invades the parathyroid widely, by an infiltrative growth (Haematoxylin & Eosin stain, ×20, (a)) (Haematoxylin & Eosin stain, ×40, (b)).

**Figure 2:**
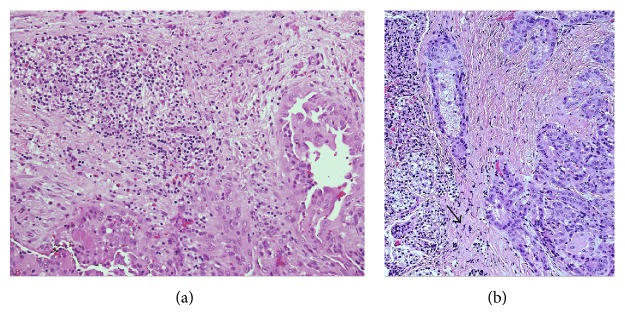
(a) Papillary thyroid carcinoma invading the parathyroid gland by a pattern A infiltration (Haematoxylin & Eosin stain, ×20). (b) Papillary thyroid carcinoma (*on the right*) invading a parathyroid gland by a pattern B infiltration. The parathyroid is located outside the thyroid capsule, which is separated from by an intervening fibrous capsule (*arrow*) (Haematoxylin & Eosin stain, ×20).

Table 1 summarizes the clinical, pathological, and follow-up data of the 10 patients included in the study. Of them, 7 (70%) were female and 3 (30%) male (female to male ratio 2.3 : 1), aged 55 ± 14 years (range 34–76 years, median age 56 years). Histological examination disclosed PTC in all patients, of whom 6 had classic PTC, 3 follicular variant PTC, and 1 tall cell variant PTC (Figures 1(a) and 1(b)). FNAB reports before surgery were Thy5 in any case. The mean PTC nodule size was 2.4 ± 0.6 cm (median 2.4 cm): neither microcarcinomas (i.e., malignant nodules smaller than 10 mm) nor multifocal PTCs were seen. The tumor extended beyond the thyroid capsule invading perithyroid soft tissues in 6 out of 10 cases; no massive extrathyroid extension involving larynx, trachea, or esophagus occurred in any patient. Lymph node metastases in the central neck (level VI) compartment were shown in 3 out of 10 patients. Parathyroid involvement by TC occurred by the following patterns: direct invasion (pattern A) in 6 cases (Figure 2(a)), extension of the primary tumor through an intervening pseudocapsule (pattern B) in 1 case (Figure 2(b)), and both pattern A and pattern B in the remaining 3 cases. Post-RAI whole body scan did not show I^131^ uptake outside the thyroid bed in any patient. A final AJCC/UICC stage I was assigned to 2 out of 10 individuals (aged <45 years) and a stage III to the remaining 8 (aged >45 years): all patients were assigned a low-risk recurrence level. The rhTSH stimulation test disclosed undetectable serum thyroglobulin and anti-thyroglobulin antibody concentrations in any case. At 5.6 ± 3-yr follow-up, all patients are alive without clinical or laboratory evidence of PTC recurrence.

The results of the literature review are reported in Table 2.

## 4. Discussion

To our knowledge, the present work represents the first study performed in Italy, and the second in Europe [7], recruiting patients with parathyroid involvement by TC from a large series of thyroidectomies. With the limits due to its retrospective design, this study sheds light on the clinicopathological features and the prognostic impact of TC spread to the parathyroid in patients submitted to thyroidectomy. Obviously, our results do not reflect the true incidence of parathyroid metastasis from TC, which is actually very hard to calculate. Indeed, in our institutions, as well as in most centers worldwide, the parathyroids closest to the TC are not routinely removed and submitted to histological examination. Thus, it is likely that their involvement by TC has been largely underestimated.

Both the thyroid and the parathyroid glands are infrequently the site of metastasis from other organs. Indeed, cytological examination of fine needle aspiration biopsy performed on thyroid nodules demonstrates a primary tumor in up to 5% of cases [2], and a secondary localization in approximately 0.07% [13]. Parathyroid malignancy of primary or secondary origin is even more uncommon, as parathyroid carcinoma occurs in less than 1% of patients with primary hyperparathyroidism [14], whereas metastases to parathyroid have been described in few reports in the literature [15–17]. Nevertheless, the few autopsy studies so far conducted in known cancer patients have reported a 0.2–11.9% incidence of secondary localization to parathyroid gland [18]. The most common primary sites of origin have been breast, blood (leukemia), skin (malignant melanoma), lung, soft tissue, and lymphoma [18, 19].

Thus, the frequency of parathyroid gland involvement in metastatic cancer is not clear, actually. The outward discrepancy between the clinical and the autopsy studies might be caused by surgeons' attitude to preserve parathyroid glands by both the anatomic and functional point of view.

With regard to clinical studies, searching PubMed for articles on this topic, only four series of adult patients have been reported as yet [4–7], describing parathyroid invasion by TC. Of these, three were conducted in Japan [4–6] and one in Greece [7]. All study, including ours, recruited a small number of patients (range 10–30) and did not add a control group. Overall, 84 patients with parathyroid involvement by TC have been so far reported and were thoroughly reviewed in this paper.

In Asian cohorts, where the only PTC histotype was studied, an incidence rate of parathyroid involvement ranging 2.2–3.9% was found. In the Greek series, the files of 1,770 thyroidectomies from patients affected by all TCs were analyzed, the incidence rate was lower at 0.5%, and 3 follicular thyroid carcinomas and 1 ATC were reported, too. This rate is very close to the 0.4% incidence demonstrated in our series, where PTC was the only TC histotype metastasizing to the parathyroid. The mismatch between the “European” and the “Asian” rates is not easy to interpret, because any attempt to speculate on the known genetic differences between the two populations clashes with the few patients included in the studies and with the fact that the three Japanese studies derive from a single institution. Of note, according to the autopsy results [18], one should expect more cases of ATC invading the parathyroids than those reported in these series. A realistic explanation should be that ATC patients are very rarely submitted to thyroidectomy due to advanced stage and absence of cleavage surgical planes. In our series of 22,310 thyroidectomies, only 15 cases of ATC were detected; of these, 11 were partial thyroidectomies performed to relieve compressive symptoms (data not shown) and, therefore, parathyroid involvement—albeit highly expected—could not be evaluated. In 2 out of the remaining 4 ATC cases, massive extrathyroid extension of the tumor involving also the parathyroids occurred; however, because patients had died, available data were too poor to include them in this study.

The majority of subjects were of female gender, with a female to male ratio ranging from 2.3 to 1 in either the Asian or our series, and 4 to 1 in the Greek one. Most of them were in their sixth-seventh decade of life. It should be mentioned that Kakudo et al. [5] demonstrated a significantly older age (mean: 60.4 yrs) in 14 patients with parathyroid involvement from PTC compared to PTC patients without parathyroid invasion (mean: 49.8 yrs). However, Tang et al. [4] enrolled more patients (20 subjects) and found a mean age lower at 52 yrs, which was essentially the same reported in the Greek [7] and in the present cohorts.

**Figure 3:**
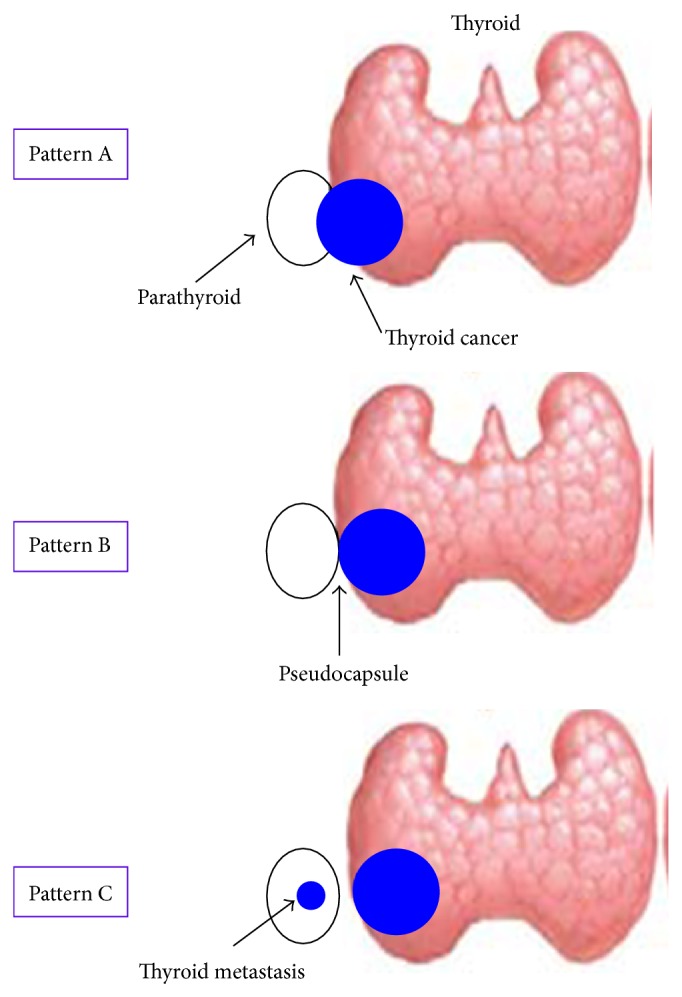
Patterns of parathyroid involvement by thyroid cancer. Pattern A: direct invasion by infiltrative growth from the primary thyroid neoplasm; Pattern B: extension of thyroid malignancy into the gland, from which cancer nests are separated by an intervening fibrous capsule (pseudocapsule); Pattern C: true metastasis within the gland, with no evidence of continuity from the primary thyroid tumour.

By the pathological point of view, parathyroid involvement may occur in three possible patterns (Figure 3): (1) direct invasion by infiltrative growth from the primary TC (pattern A); (2) extension of TC into the gland, from which cancer nests are separated by an intervening fibrous capsule (pseudocapsule) (pattern B); (3) true metastasis within the gland, with no evidence of continuity from the primary TC (pattern C). Pattern A and pattern B represent by far the most frequent findings in the literature, occurring in >90% of studied individuals. Interestingly, our patients carried the pattern A and the pattern B of parathyroid involvement, alone or in combination. No case of true metastasis (pattern C) from TC was shown in our series.

A matter of uncertainty in the literature, at present, is if the parathyroid involvement by TC should be considered an important risk factor for recurrence and survival or, conversely, if it should be regarded as an event without any clinical and prognostic impact. Obviously, thyroidectomy-based studies are not designed to meet this point, and perspective case-control study protocols are recommended to this aim. That does not prevent us from discussing the following. In our series, one patient with the aggressive tall cell variant PTC was included, lymph node metastases were detected in 3 out of 10 patients, and invasion of perithyroid soft tissues occurred in most cases. Despite this, unexpectedly, all patients were disease-free with undetectable serum thyroglobulin concentrations after a mean 5.6-yr follow-up period. Interestingly, massive extrathyroid extension of TC was not observed in any case. On the contrary, in Kakudo et al.'s study [5], parathyroid involvement by PTC was associated to either advanced stage of disease or 10-yr disease-free survival reduced at 80.8%, mainly in patients older than 55 yrs. Chrisoulidou and coworkers [7] reviewed the files of 10 patients and discovered soft tissue invasion in 9, and lymph node and distant metastases in 2 subjects, of whom one was a 17-yr old boy. Ito and colleagues [6] demonstrated a significantly worse prognosis in patients with massive extrathyroid extension, independently of parathyroid involvement; really, TC extension to parathyroid* per se* did not impact at all on 10-yr disease-free survival, which exceeded 95% where minimal tumor extension occurred. On multivariate analysis, further predictors of poor prognosis other than massive extrathyroid extension were age > 55 yrs, stage N1b, and primary tumor size > 4 cm. Based on these results, the authors concluded that “extension to the parathyroid gland corresponds to minimal extrathyroid extension for PTC.” Thus, they confirmed the fairness of pTNM parameters on which the AJCC/International Union against Cancer (AJCC/UICC) classification system of thyroid malignancy is currently based [11], particularly where it classifies as pT3 a “tumor of any size extending beyond the thyroid capsule to invade subcutaneous soft tissues, larynx, trachea, esophagus, or recurrent laryngeal nerve.” Furthermore, in accordance with current ATA guidelines [12], TC subjects with minimal extension to perithyroid soft tissues and concomitant parathyroid metastasis should be regarded as low-risk, as was the case of our patients.

In conclusion, limited to thyroidectomy series, our results and literature data suggest that parathyroid involvement by TC has a 0.4–3.9% incidence rate; mainly affects women in their sixth-seventh decade of life; is associated to a good prognosis, unless massive extrathyroid extension of TC occurs.

## Figures and Tables

**Table 1:** Personal and histological characteristics of the 10 patients recruited in the present study.

	Patient 1	Patient 2	Patient 3	Patient 4	Patient 5	Patient 6	Patient 7	Patient 8	Patient 9	Patient 10
Sex	F	M	F	F	F	F	M	F	F	M
Age (yrs)	58	44	54	76	34	38	66	65	68	47
Primary tumor histotype	FVPTC	Tall cell PTC	Classic PTC	Classic PTC	FVPTC	Classic PTC	Classic PTC	FVPTC	Classic PTC	Classic PTC
Primary tumor size (cm)	1.5	3.3	1.6	2.3	3	2.3	1.9	2.8	3.2	2.5
Extrathyroid invasion of tissues other than parathyroid	No	Yes	No	Yes	No	Yes	No	Yes	Yes	Yes
Lymph node metastases	Yes	Yes	No	No	No	Yes	No	No	No	No
Distant metastases	No	No	No	No	No	No	No	No	No	No
Patterns of parathyroid involvement	Pattern B	Pattern A	Pattern A + B	Pattern A	Pattern A	Pattern A + B	Pattern A	Pattern A	Pattern A	Pattern A + B
Stage^*^	III (pT3N1aM0)	I (pT3N1aM0)	III (pT3N0M0)	III (pT3N0M0)	I (pT3N0M0)	I (pT3N1aM0)	III (pT3N0M0)	III (pT3N0M0)	III (pT3N0M0)	III (pT3N0M0)
Recurrence	No	No	No	No	No	No	No	No	No	No
Follow-up time (yrs)	3	10	6	4	5	2	9	8	1	7

FVPTC: follicular variant of papillary thyroid carcinoma; PTC: papillary thyroid carcinoma; Pattern B: extension of thyroid malignancy into the parathyroid, in which cancer nests are separated from by a pseudocapsule; Pattern C: true thyroid cancer metastasis within the parathyroid, with no evidence of continuity from the primary thyroid tumor. ^*^Based on AJCC/International Union against Cancer (AJCC/UICC) classification system [11].

**Table 2:** Epidemiological, clinical, pathological, and prognostic features of patients with parathyroid involvement by thyroid cancer: review of the literature.

	Tang et al., 2002 (Ref. [4])	Kakudo et al., 2004 (Ref. [5])	Ito et al., 2009 (Ref. [6])	Chrisoulidou et al., 2012 (Ref. [7])	Present study	Total/conclusions
Rate of parathyroid involvement in thyroid cancer patients	2.2%	3.9%	1.6%	0.5%	0.4%	Range: 0.4–3.9%
Number of patients enrolled	20	14	30	10	10	84
Sex	14 F, 6 M	10 F, 4 M	Not reported	8 F, 2 M	7 F, 3 M	F to M ratio: 2.3–4 to 1
Mean age (yrs)	52	60.4	Not reported	52.2	55	Range: 52–60.4
Primary tumor histotype	20 PTC	14 PTC	30 PTC	6 PTC 3 FTC 1 ATC	10 PTC	80 out of 84 cancers are PTC
Primary tumor size	>1 cm 14 ≤1 cm 6	2.5 cm (mean)	>1 cm 22 ≤1 cm 8	2.5 cm (mean)	2.4 cm (mean) >1 cm 10	Microcarcinomas are uncommon
Extrathyroid invasion of tissues other than parathyroid	10 yes 10 no	13 yes 1 no	8 yes 22 no	9 yes 1 no	6 Yes 4 no	46 yes 38 no
Lymph node metastases	17 yes 3 no	13 yes 1 no	1 yes 29 no	5 yes 5 no	3 yes 7 no	39 yes 45 no
Distant metastases	1 yes 19 no	2 yes 12 no	0 yes 30 no	2 yes 2 no	0 yes 10 no	5 yes 79 no
Patterns of parathyroid involvement	Pattern A 15 Pattern B 3 Pattern C 2	Pattern A 10 Pattern B 3 Pattern C 1	Not reported	Pattern A + B 9 Pattern C 1	Pattern A 6 Pattern B 1 Pattern A + B 3	Pattern A + B 90–100% Pattern C 0–10%
Stage^*^	Not reported	I/II 2 III/IV 12	pT3 30/30	I/II 3 III/IV 7	I 2 III 8	—
Recurrence	Not reported	2 yes 12 no	1 yes 29 no	Not reported	0 yes 10 no	Most cases do not recur
10-yr disease free survival rate	Not reported	80.8%	>95%	Not reported	N/A	>80% in the reported cases

ATC: anaplastic thyroid carcinoma; F: female; FTC: follicular thyroid carcinoma; M: male; N/A: not applicable; PTC: papillary thyroid carcinoma; Pattern A: direct invasion by infiltrative growth from the primary thyroid carcinoma to parathyroid; Pattern B: extension of thyroid malignancy into the parathyroid, in which cancer nests are separated from by a pseudocapsule; Pattern C: true thyroid cancer metastasis within the parathyroid, with no evidence of continuity from the primary thyroid tumor; Ref.: reference. ^*^Based on AJCC/International Union against Cancer (AJCC/UICC) classification system [11].
